# Exploratory space-time analysis of dengue incidence in Trinidad: a retrospective study using travel hubs as dispersal points, 1998–2004

**DOI:** 10.1186/1756-3305-7-341

**Published:** 2014-07-22

**Authors:** Karmesh D Sharma, Ron S Mahabir, Kevin M Curtin, Joan M Sutherland, John B Agard, Dave D Chadee

**Affiliations:** 1 Ministry of Health, 63 Park Street, Port of Spain, Trinidad, West Indies; 2 Department of Geography and Geoinformation Science, George Mason University, Fairfax, Virginia, USA; 3 Department of Life Sciences, The University of the West Indies, St. Augustine, Trinidad, West Indies

**Keywords:** Dengue, Space-time analysis, Travel hubs, Hot spots, Dengue epidemiology, Cluster analysis, Control programs, Trinidad

## Abstract

**Background:**

Dengue is an acute arboviral disease responsible for most of the illness and death in tropical and subtropical regions. Over the last 25 years there has been increase epidemic activity of the disease in the Caribbean, with the co-circulation of multiple serotypes. An understanding of the space and time dynamics of dengue could provide health agencies with important clues for reducing its impact.

**Methods:**

Dengue Haemorrhagic Fever (DHF) cases observed for the period 1998–2004 were georeferenced using Geographic Information System software. Spatial clustering was calculated for individual years and for the entire study period using the Nearest Neighbor Index. Space and time interaction between DHF cases was determined using the Knox Test while the Nearest Neighbor Hierarchical method was used to extract DHF hot spots. All space and time distances calculated were validated using the Pearson r significance test.

**Results:**

Results shows that (1) a decrease in mean distance between DHF cases correlates with activity leading up to an outbreak, (2) a decrease in temporal distance between DHF cases leads to increased geographic spread of the disease, with an outbreak occurrence about every 2 years, and (3) a general pattern in the movement of dengue incidents from more rural to urban settings leading up to an outbreak with hotspot areas associated with transportation hubs in Trinidad.

**Conclusion:**

Considering only the spatial dimension of the disease, results suggest that DHF cases become more concentrated leading up to an outbreak. However, with the additional consideration of time, results suggest that when an outbreak occurs incidents occur more rapidly in time leading to a parallel increase in the rate of distribution of the disease across space. The results of this study can be used by public health officers to help visualize and understand the spatial and temporal patterns of dengue, and to prepare warnings for the public. Dengue space-time patterns and hotspot detection will provide useful information to support public health officers in their efforts to control and predict dengue spread over critical hotspots allowing better allocation of resources.

## Author summary

The increased geographic spread and intensity of dengue is due to numerous factors including, increased urbanization, human migrations and air travel, flooding and global warming. In the Caribbean, outbreaks continue to occur with hyperendemic occurrence of the disease. This is mainly due to the use of reactive programs and limited resources available to control the disease. Using the island of Trinidad as a case study, we show that higher rates of infection occur in areas with a history of dengue incidence. Also, a general pattern in the movement of dengue cases is found leading up to and transitioning away from an epidemic occurrence, and associated with the locations of transportation hubs. These findings can be used to contain the disease in a more efficient and effective manner. Also, few studies have examined the space and time relationship of dengue incidents at local scales in the Caribbean islands. Other islands can adopt the approach used to better allocate resources and understand the disease. This information can then be used to gain regional perspective and understanding about the spatio-temporal persistence of dengue in the Caribbean.

## Background

The concept that human movement enhances the spatial distribution of dengue fever is not new and has been demonstrated along busy routes of human traffic in Asia [1-3] and more recently in the Americas [4,5]. In fact wherever people congregate in dengue infested countries whether at home, in church with friends and at relatives and festivals, the likelihood of man-vector-virus contact increases thereby enhancing disease transmission [6]. Studies conducted by Chao et al. [7] modeled the role human movement played in the spatial spread of dengue and suggested that dengue cases which clustered in space and time were probably due to the short flight range of the *Ae. aegypti* mosquito [8]. This clustering of cases and the concept of “casa segura” demonstrate the role human movement plays in dengue spatial transmission [1-3,9,10]. Similar studies have been recently conducted in India [11], Peru [12], Cambodia [10] and Puerto Rico [4].

Dengue is an acute tropical viral disease caused by one of four serotypes, Dengue-1, Dengue-2, Dengue- 3, and Dengue-4 (DEN-1, DEN-2, DEN-3, and DEN-4) transmitted by Aedes mosquitoes; *Aedes aegypti* (*L*.) and *Aedes albopictus* (Skuse) [13]. Of the two species of Aedes mosquitoes, *Ae. aegypti* has been identified as the principal vector for much of the morbidity and mortality associated with dengue [14]. Adult female *Ae. aegypti* acquire the virus by biting an infected person during the viremic phase, which usually lasts for four to five days, but may last up to 12 days [15]. The virus is then transmitted to non-infected persons via bites from infected mosquitoes. Clinical symptoms of infected persons vary from a mild, self-limited fever, which can be undiagnosed or misdiagnosed, to severe illness with acute hemorrhagic manifestations such as Dengue Hemorrhagic Fever (DHF) or Dengue Shock Syndrome (DSS) [16,17]. These severe forms of the disease are associated with several factors, including the immunity status of hosts, disease strain [18], age and genetic predisposition [19].

Over the last 25 years the overall incidence of dengue in developing regions of the world, including the Caribbean region [20,21] has increased with circa 40% of the world’s population at risk of infection with dengue [7]. It is now considered the most frequent arboviral disease in the world, with an estimated annual 390 million cases of dengue fever (DF), 250,000 cases of DHF, 25,000 deaths per year and 93 million asymptomatic cases [7,22]. Within Latin America and the Caribbean region, over 908,926 cases of DF and DHF have been reported up to 2008 [22]. In Trinidad, dengue outbreaks continue to occur despite conventional vector control efforts with the situation made worst by the absence of suitable vaccines for the disease.

An important issue in monitoring communicable diseases and planning intervention is to understand the spatial and temporal patterns of disease. This information can provide clues to understanding the dynamics of disease spread, especially the detection of spatial and space-time clustering of cases and identifying high risk areas and times, where disease surveillance and control should be targeted. In this study several methods are used for spatial and temporal analysis of DHF cases in Trinidad for the period 1998–2004. The Nearest Neighbor Index (NNI) and the Nearest Neighbor Hierarchical Clustering (NNHC) methods have been widely used in exploring first and second order spatial point processes [23]. The NNI is used to detect the presence of spatial clustering while NNHC is used to map the extent of the spatial clustering if present. Recent studies have demonstrated the utility of the NNI for detecting the presence of spatial clustering in dengue cases [24,25] while the NNHC was used to identify high mortality concentrations of yellow fever for each month in 1878 representing an epidemic year of the disease in New Orleans [26]. The NNHC clustering is particularly applicable if the nearest neighbor distance is believed to be of relevance to the spatial and temporal distribution of point features [27]. Work conducted in the County of St Patrick, Trinidad showed dengue cases closely clustered together and suggested that this clustering was possibly due to the short flight range of *Ae. aegypti*[28], thus making NNI and NNHC suitable methodologies for the current study. The present study was conducted to investigate the impact of different road networks on the spatio-temporal distribution of dengue fever cases associated with the locations of transportation hubs in Trinidad, West Indies.

## Methods

### Digital datasets

All digital datasets collected or created were represented as thematic layers and converted to a common geographic coordinate system, UTM Zone 20 N with an earth model of WGS 1984 to support uniform analysis of the data. Resulting datasets were then analyzed using Geographic Information System (GIS) software. Geographic datasets used in this study were collected from the Department of Geomatics Engineering and Land Management, University of the West Indies, St. Augustine, Trinidad.

### Case data

A definition of a confirmed DHF case was provided by the Ministry of Health, Trinidad and Tobago. This definition was framed as persons (child or adult) having the symptoms: temperature of 38°C or higher for 5 days, accompanied by headache, myalgia, and other non-specific clinical presentations [29]. These persons were closely monitored for signs of haemorrhage. All suspected cases of DHF were laboratory confirmed by virus isolation, detection of specific IgM antibody and/or seroconversion [29]. Records representing confirmed DHF cases for 1998–2004 were collected from the Ministry of Health. These contained street addresses of persons confirmed to be infected by the disease. The years 1998–2004 were specifically chosen since they represent data which could be obtained for Trinidad, along with presenting an opportunity to examine the transition between two dengue epidemic years (1998 and 2002) on the island. This study was approved by the Ethics Committee of the South West Regional Health Authority, Ministry of Health, San Fernando, Trinidad, West Indies.

### Address geocoding and spatial distribution

A GIS is a tool that incorporates geographical features with tabular data in order to map, analyze, and assess real-world problems. It has been used extensively to study diseases, relating spatial patterns to geographic variations in health risks [25]. The names of all dengue cases were deleted and each subject given a unique identifier number before data entry was conducted to safeguard the identity of patients. However, a master sheet was kept in a secure location with access available only to the Principal investigator. In this study, the street addresses of confirmed DHF cases were located on a GIS road layer of Trinidad. Successfully located cases were then plotted as point features; creating a new GIS layer representing point locations of DHF cases in Trinidad. Due to insufficient information, not all cases could be geocoded e.g. exact addresses were missing as well as other demographic information. These cases were excluded from our analysis.

### Cluster analysis

#### Spatial clustering

Being an island, Trinidad imposes a natural boundary on both human and mosquito populations. Trinidad is roughly rectangular in shape with most people living in areas along the coastline. We therefore used a rectangular bounding box around the observations as the area to be used in calculations of clustering. Although one could argue that there are some internal uninhabited areas that could be removed from the analysis, doing so would only serve to strengthen the level of clustering described below.

Spatial clustering was determined using the Nearest Neighbor Index [30,31]. This was done for each year followed by an application for all years. The NNI measures the degree of spatial dispersion in the distribution based on the minimum of the inter-feature distances. It compares the distances between nearest points (Nearest Neighbor Distance) and distances that would be expected on the basis of chance (Random Distance) as shown in eq. 1.

(1) $$\mathrm{NNI}=\;\frac{\mathrm{NND}}{\mathrm{RD}}$$

where NNI is the nearest neighbor index, NND is the mean nearest neighbor distance and RD is the random distance. As explained in [31], NND is calculated by first calculating the distance between each feature and every other feature. The minimum distance (representing the distance to the closest feature) for all features is then averaged by dividing by the total number of features as shown in eq. 2.

(2) $$\mathrm{NND}=\sum_{i=1}^{N}\left[\frac{\mathrm{Min}\left(d_{\mathrm{ij}}\right)}{N}\right]$$

where *Min*(*d*_*ij*_) is the distance between each feature and its nearest neighbor and N is the number of features in the distribution. The calculation of RD is shown in eq. 3 and its numerical proof presented in [31].

(3) $$\mathrm{MD}=\;\frac{1}{2\sqrt{\rho }}$$

where *ρ* represents the density of the observed distribution calculated as the total number of features divided by the area of the region containing the features. Also proposed by [31] is a Z-test to indicate whether the observed mean nearest neighbor distance was significantly different from the random distance as shown in eq. 4.

(4) $$Z=\;\frac{\mathrm{NND}-\mathrm{RD}\;}{SE_{\mathrm{NND}}}$$

where *SE*_*NND*_ is the standard error of the mean nearest neighbor distance and is calculated as shown in eq. 5 and its numerical proof presented in [31].

(5) $$SE_{\mathrm{NND}}=\;\frac{0.26136}{\sqrt{\mathrm{N\rho}}}$$

where N represents the total number of features and *ρ* is the density. A Z-value indicating 95% confidence (values between −2.58 and 2.58 standard deviations) was used as a measure of significance for each calculated NNI that indicates clustering of features in 2D space. Calculations were done for each year and across all years respectively.

To map the extent of clustering, identifying hotspots of dengue incidence, the NNHC method was used. NNHC identifies cases that are spatially close to each other. Disease cases are placed into a particular cluster based on rules. In this case, a mean random distance threshold was used (as calculated for NNI). Also, the size of a cluster was established as an area where a minimum of 10 cases were close to each other in space. The researchers recognize that this threshold is subjective, however based on the available literature there is no fixed size for what constitutes a cluster. Only points that fit both criteria (closer than the threshold and belonging to a group having the minimum number of points) are clustered at the first level (first-order clusters). This method then conducts subsequent clustering to produce a hierarchy of clusters where the centroids of first order clusters are then tested using the same rules for clustering (second order) and this process repeats until all first order clusters eventually converge to a single cluster or a rule is broken. The resultant clusters were represented as ellipses around contributing points using a standard deviation value of 1. The value of 1 was used as suggested by [23] since larger values can create an exaggerated view of the underlying cluster.

#### Spatio-temporal clustering

Spatio-temporal clustering was tested using the Knox test. It was first done for each year and then applied to all years. It is one of the most widely used statistical techniques for testing space-time interaction [32]. The time (date of onset subtracted from the year 1900) and geographical location of each case was noted, and for each possible pair of cases, the distances between them were calculated both in terms of space and time. The distance between points is divided into two groups, close in distance and not close in distance, and the time interval is also divided into two groups, close in time and not close in time. To calculate close in distance and close in time thresholds the mean value for spatial distance and temporal distance (represented in days) between all points was used. Points falling within these thresholds were considered close in distance or close in time based on which threshold was used. Points outside these thresholds were considered either not close in distance or close in time. The actual number of pairs that falls into each of the four areas is then compared to the expected number if there was no relationship between closeness in distance and closeness in time. The expected number of pairs in each area, under strict independence between distance and the time interval, is obtained by the cross-products of the columns and row totals. The relationship between observed and expected closeness in space and time is shown in Tables 1 and 2[33].

**Table 1:** Logical structure of Knox index for observations

	**Close in time**	**Not close in time**	
Close in distance	O_1_	O_2_	S_1_
Not close in distance	O_3_	O_4_	S_2_
	S_3_	S_4_	N

**Table 2:** Logical structure of Knox index for expected values

	**Close in time**	**Not close in time**
Close in distance	E_1_	O_2_
Not close in distance	E_3_	O_4_

where N = O_1_ + O_2_ + O_3_ + O_4_

$$S_{1}=O_{1}+O_{2}$$

$$S_{2}=O_{3}+O_{4}$$

$$S_{3}=O_{1}+O_{3}$$

$$S_{4}=O_{2}+O_{4}$$

where E_1_ = (S_1_ * S_3_)/N

$$E_{2}=\left(S_{1}*S_{4}\right)/N$$

$$E_{3}=\left(S_{2}*S_{3}\right)/N$$

$$E_{4}=\left(S_{2}*S_{4}\right)/N$$

A one-tailed Chi-squared statistic is then used to accept or reject the null hypothesis based on a 95% confidence value. A rejection of the null hypothesis indicates that there is clustering in both space and time. Because of the interdependency of cases with respect to space and time, a Monte Carlo simulation of Chi-squared values for the Knox Index under spatial randomness was used. Recent work has explored the nature of the distribution of the Knox statistic in comparison to known distributions like the Chi-squared distribution [32]. In general, Monte Carlo simulation is the preferred method of generating a distribution of values for a known area and a known phenomenon. For each routine (of 1000 routines in total) M pairs of distance and a time interval is randomly chosen from the minimum and maximum values for distance and time in the dataset. M represents the number of pairs in the dataset (M = N * [N-1]/2). These values are used to calculate the Knox Index and the Chi-square test. The mean value for both distance and time was calculated.

## Results

### Geocoded dengue cases

Table 3 shows the number of DFH cases geocoded each year for the period 1998 to 2004. From a total of 1212 confirmed DHF cases, 825 cases (68%) were actually geocoded and were used in the analysis. The geocoded cases for 2002 (which was also an epidemic year) represented only 44% (197 cases) of the original number of cases collected. The low number of geocoded cases for this year was primarily due to poor documentation or poor reporting of dengue cases to the Ministry of Health in Trinidad.

**Table 3:** Number of dengue cases geocoded by year

**Year**	**Number of collected cases**	**Number of geocoded cases (and percentage)**
1998	207	156 (75)
1999	56	47 (84)
2000	146	129 (88)
2001	178	149 (84)
2002	448	197 (44)
2003	148	130 (88)
2004	29	17 (59)
*Total*	*1212*	*825* (*68*)

### Spatial distribution of dengue

**Figure 1:**
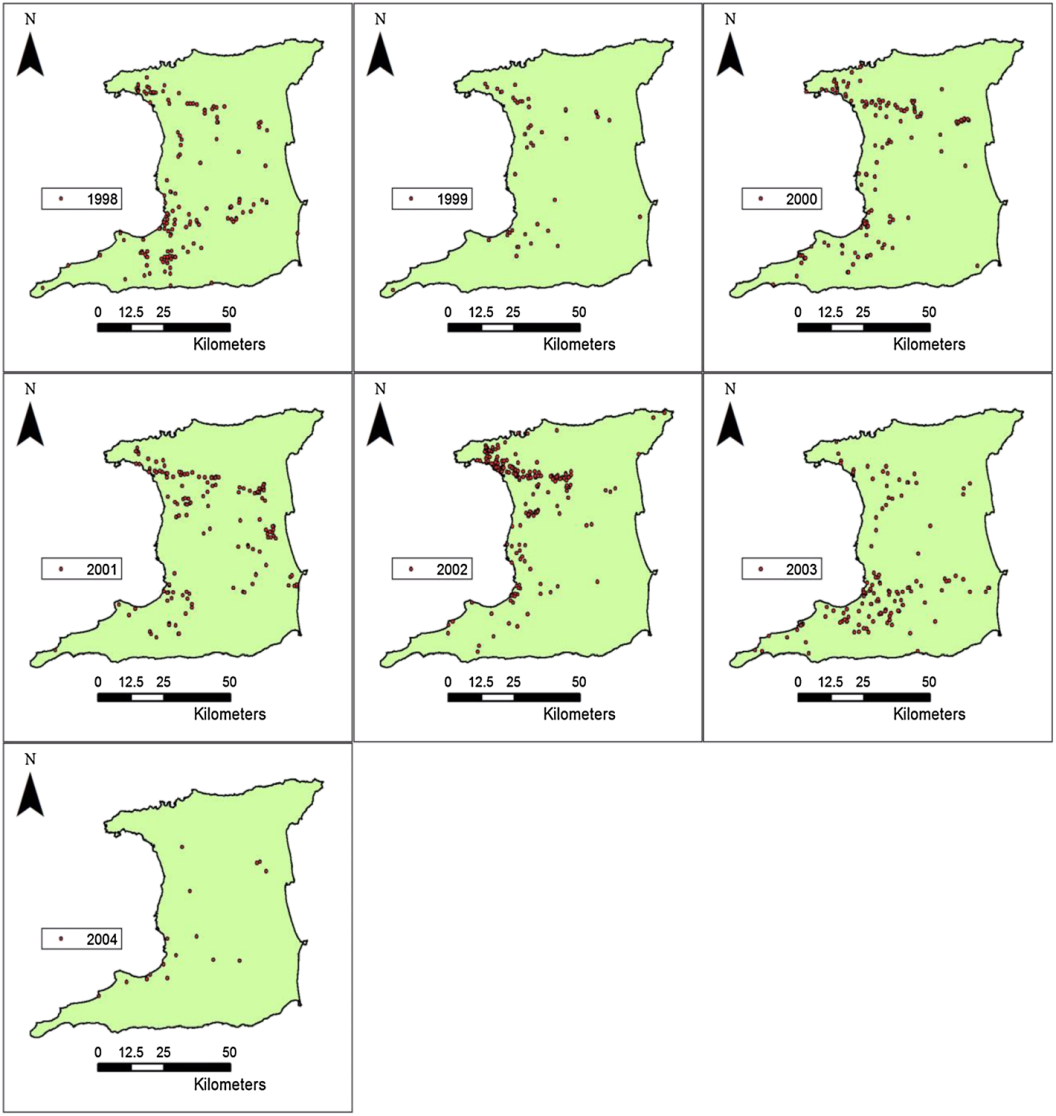
Spatial distribution of dengue hemorrhagic fever cases in Trinidad, 1998–2004.

Figure 1 shows the spatial distribution of DHF cases by year over the 7 year study period. These results show the transition between the epidemic years 1998 and 2002, with a significant (G = 57.4; d.f. 6; P < 0.03) decline in the number of cases immediately after 1998. This was followed by the cases becoming more dispersed or scattered, after which a significant (G = 87.2; d.f.6; P < 0.01) increase in the number of cases occurred towards 2002 with a high density of cases in space (see Figure 1). Although the results showed where dengue cases were clustered, it was impossible to demonstrate the full pattern because all DHF cases, or at least a substantial quantity, were not geocoded for 2002. However, similar to activity soon after the epidemic in1998, immediately after 2002, a significant decline (G = 54.2; d.f. 6; P < 0.02) in the number of cases is detected.

### Spatial cluster analysis

#### Nearest neighbor analysis

**Figure 2:**
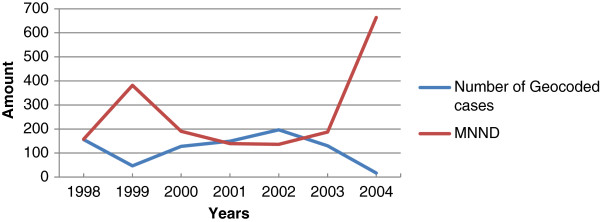
Geocoded DHF cases and the MNND for individual years 1998–2004 in Trinidad.

Table 4 shows the result of the Nearest Neighbor analysis with spatial clustering occurring for individual years 1998–2003 but absent during 2004. During 2004 only 17 (of 29) DHF cases were geocoded, which represented a year with a significant decline (P < 0.02) in DHF cases with very dispersed spatial distribution (a mean value of 6638.1 m according to Table 4), (see Figure 1 and Table 3). Table 4 shows that DHF cases were very densely clustered across years in space (mean of 598 m), suggesting a higher probability of infection in an area with a history of infection.Figure 2 shows the number of geocoded cases and the Minimum Nearest Neighbor Distance (MNND) plotted for individual years. Values for MNND in Figure 2 were scaled by dividing the original value by 10 to aid in visual comparative analysis. Figure 2 shows a reversed trend when the number of DHF cases and MNND were compared, that is, as the number of DHF cases increases, the mean distance between cases decreases. In addition, there was significant (P < 0.03) dispersion between cases soon after an epidemic has occurred, as indicated by the slope of the MNND line just after 1998 and 2002. It is expected that with more geocoded cases for 2002 the spatial dispersion (MNND) would have been significantly greater in the following years. The high spatial dispersal pattern observed represents the mean distance found between cases after outbreaks.

**Table 4:** Nearest neighbor analysis for DHF cases in Trinidad

**Year**	**P-Value**	**Mean nearest neighbor distance (m)**
1998	0.0001	1576.9
1999	0.0001	3815.8
2000	0.0001	1902.5
2001	0.0001	1396.7
2002	0.0001	1362.3
2003	0.0001	1877.5
2004	Not significant	6638.1
All years	0.0001	598.0

#### Nearest neighbor hierarchical clustering

**Figure 3:**
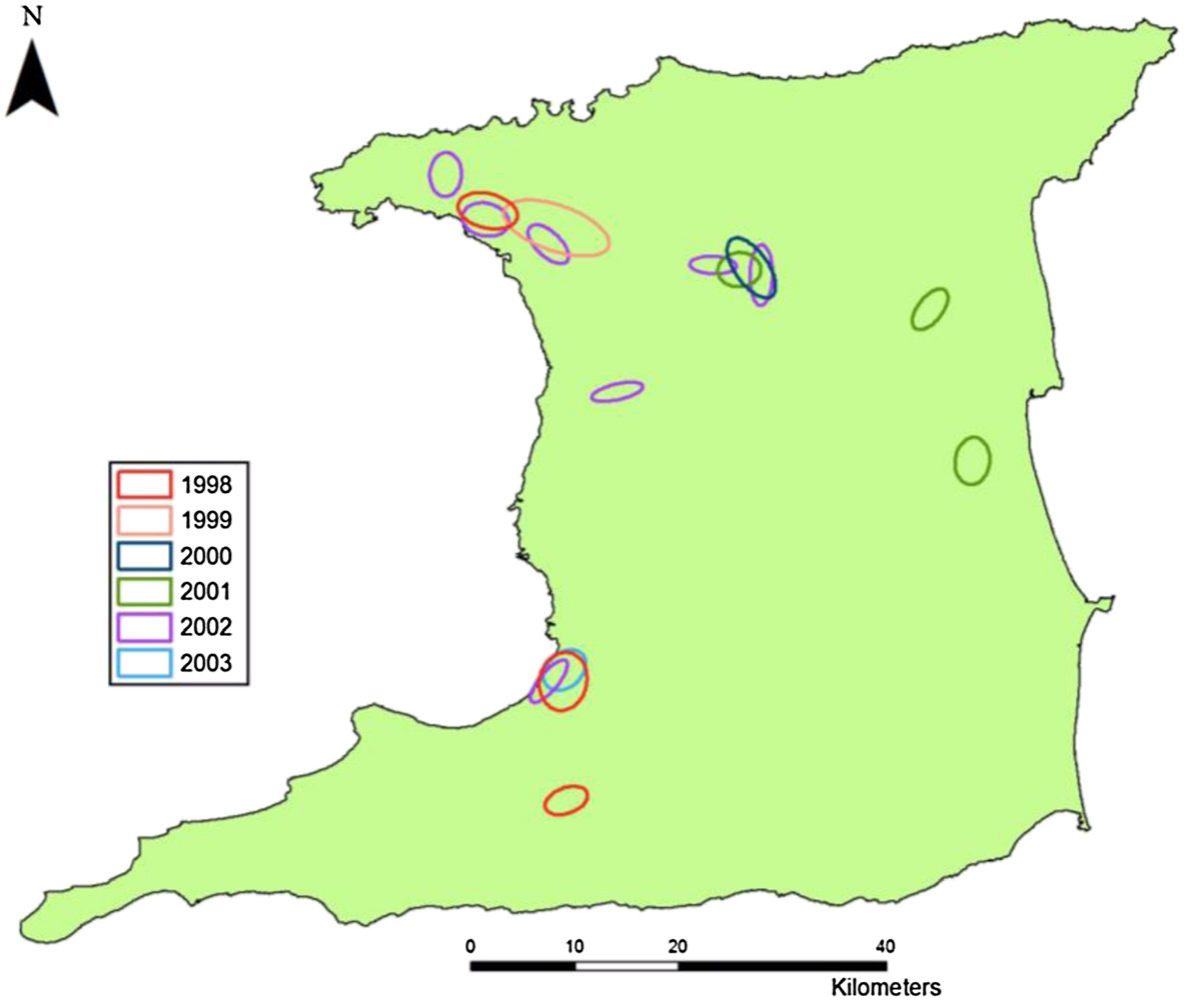
Nearest neighbor hierarchical clustering.

**Figure 4:**
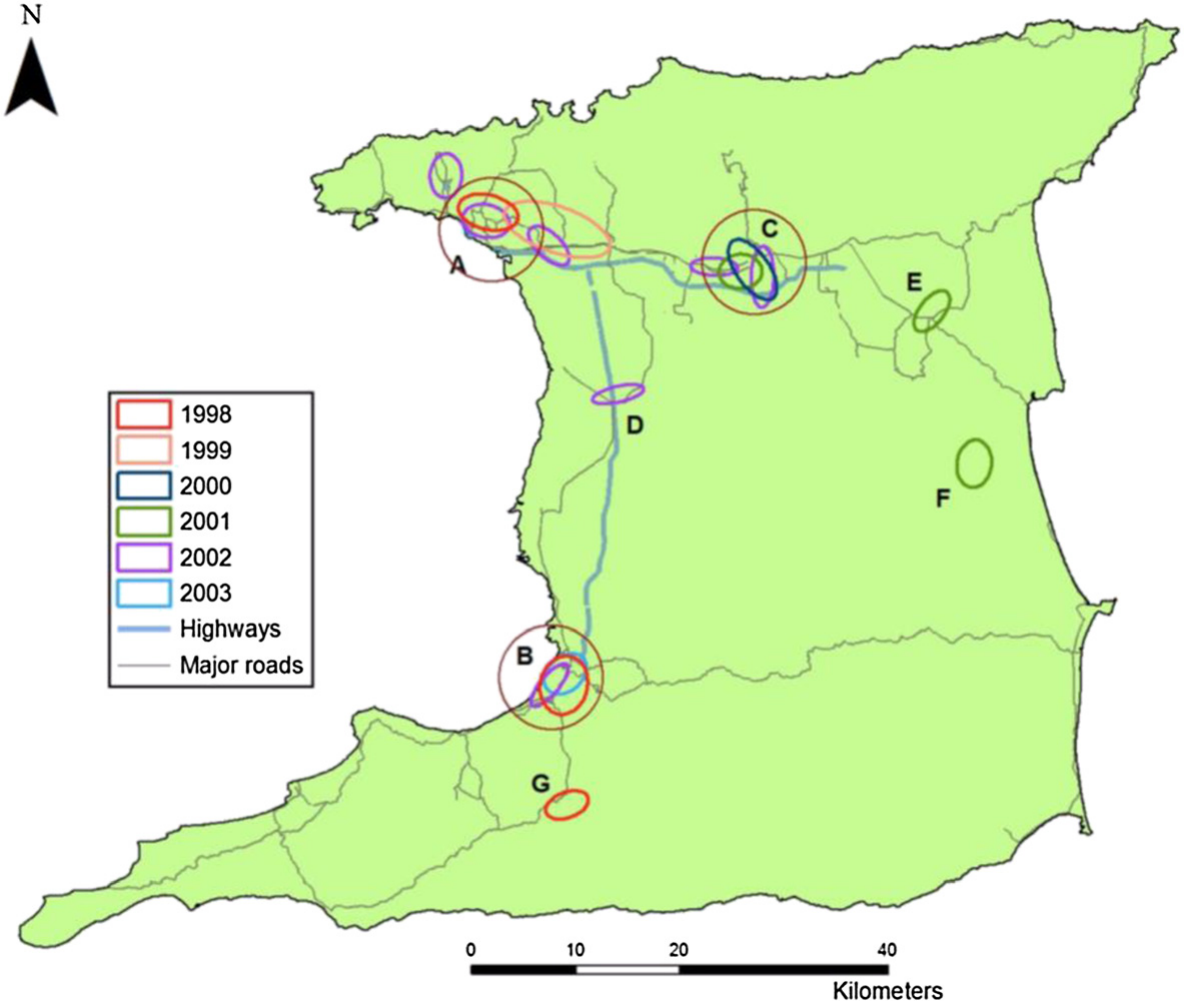
Transportation hubs and dengue clusters in Trinidad (A-Port of Spain, B-San Fernando, C-Arima, D-Chaguanas, E-Sangre Grande, F-Biche, and G-Penal).

The hierarchical ellipses show the identified hotspots of dengue cases for individual years 1998 to 2003 (Figure 3), and represent the spatial distribution of DHF cases detected using only the first order clusters. The years 1999 and 2004 are not shown in this figure since there were no clusters identified based on the criteria set for determining the presence of clustering (a minimum of 10 points and using a mean random distance threshold based on the dataset as explained in the previous section). Figure 3 shows three main regions of clustering indicated in Figure 4 by regions A, B and C (encircled by red ellipses) followed by 4 smaller regions D, E, F and G. Region A and B are the main city capitals in Trinidad, Port of Spain and San Fernando respectively. Region C represents an equally important major town of Arima, which is smaller in size than to the identified cities in regions A and B.Figure 4 shows hierarchical clustered regions superimposed onto a road network of Trinidad. This figure clearly shows the relationship between dengue clusters and areas within and around transportation hubs on the island. Regions A, B and C represent the island’s major transportation hubs between major cities while region E (town of Sangre Grande) represents another transportation hub which links most of the North Eastern part of the island to highways and by extension, the major cities in Trinidad. Region G (Penal) represents a smaller town near the suburban part of the island while region F represents an even smaller town in the rural part of Trinidad. Figure 4 also reveals a distinct movement of dengue clusters between types of transportation hubs and epidemic years with clustering occurring in and around major cities during an epidemic (through major transportation hubs). Following this, the results show migration towards more rural settings, transitioning away from an epidemic and towards areas with smaller transportation hubs (and nearer to smaller towns) on the island. The reverse relationship is seen transitioning towards an epidemic year.

### Space-time analysis

**Figure 5:**
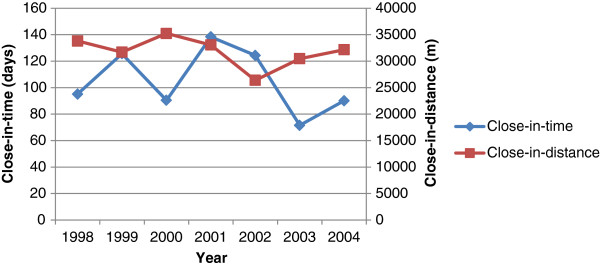
Close in distance and close in time values for individual years dengue occurred in Trinidad.

Using the Knox test the results showed a significant number of dengue cases were clustered in space (G = 76.7; d.f.6; P < 0.05) and time (G = 67.9; d.f. 6; P < 0.04) within and across years (∑^2^ = 345.97 d.f. 10; < 0.05) (see Table 3). Table 5 shows that the clustering time between years is approximately 727 days (about 2 years) and represents the transition between epidemic years 1998 and 2002, where in 2000 (2 years after) cases began appearing once more and transitioned towards the epidemic year 2002. Figure 5 shows the 'close in distance' and 'close in time' values for individual years. Values for 'close in distance' in Figure 5 were scaled by dividing the original value by 1000 to aid in visual comparative analysis.Figure 5 shows a reversed relationship between 'close in distance' and 'close in time' variables with the exception of the transition between years 2001 to 2002. This is possibly due to the fact that only 44% of the DHF cases were geocoded in 2002, but overall the results show when the number of days decreases between clustering in time the distance between events gets larger and vice versa. This relationship suggests that when an outbreak of disease occurs within short time periods there is a faster rate of distribution of the disease across space (see Figure 5).

**Table 5:** **Space**-**time analysis of DHF cases in Trinidad** (**1998**–**2004**)

**Year**	**P**-**Value**	**Close in time (days)**	**Close in distance (m)**
1998	0.0001	95.1	33799.0
1999	0.0001	125.6	31692.9
2000	0.0001	90.4	35235.9
2001	0.0001	138.3	33049.1
2002	0.0001	124.3	26386.1
2003	0.0001	71.5	30467.4
2004	0.0010	90.1	32158.2
All years	0.0001	727.0	506774.5

## Discussion

In the 1970s and 1980s dengue outbreaks occurred in the Caribbean region at 8–10 year intervals when new serotypes were introduced [6]. However, this epidemiological pattern changed with the outbreak of dengue in Cuba in 1981 when a DEN-2 outbreak was followed by a DEN-1 episode [34]. However, in 1994, the fourth serotype DEN-3 was introduced from Southeast Asia to the Caribbean region after an absence of 17 years. This introduction has increased the frequency of epidemics from 8 to 10 years to 3 to 5 year intervals, especially in Trinidad [35]. The present results show the frequency of dengue outbreaks at approximately 727 days or circa every 2 years which suggest that the transition between outbreaks have further shortened and the frequency of dengue outbreaks reduced from every 3 to 5 years to every 2 to 3 years. This close frequency of outbreaks is cause for concern for DF/DHF in the Caribbean region and especially in Trinidad can emerge as a major public health problem with an increase in DHF/DSS burden of disease and the accompanying impacts on morbidity and mortality rates, DALYs and economic cost ([35,36]).

In Trinidad DF/DHF outbreaks occur during the wet season and are usually associated with thousands of people seeking medical treatment and hospitalization. Although not reported in the results section of this paper, this study found most reported cases for dengue recorded between June and November, coinciding with the temporal periodicity of dengue epidemics during the wet season on the island. This pattern is very similar to that reported previously for Trinidad [28] and Puerto Rico [37] but is different from that observed in Paraguay and Argentina [38]. In the latter two locations, dengue transmission occurs during the first half of the year in the southern hemisphere and during the second half of the year in the northern hemisphere, which is equivalent to the wet season for both hemispheres when temperature, humidity and precipitation are elevated.The spatial and temporal patterns of reported cases of DHF in Trinidad were analyzed using the minimum nearest neighbor distance method showed that DHF cases were clustered in both space and time within and across years (Figure 3). These results show that during an epidemic year, the number of DHF cases significantly (P < 0.02) increases and then significantly (P < 0.02) declines with a transition away from the epidemic. These results suggest that as herd immunity increases, that is as the population at risk becomes infected and then immune to the circulating dengue serotype, the susceptible population size is reduced and transmission declines. Therefore, during this time, the dengue virus and by extension DHF cases tend to occur away from intense spatial clustering towards greater spatial distribution (see Figure 3). Interestingly, the reverse effect is seen during an epidemic year, however when the number of days decrease between clustering in time, the distance between events gets larger and vice versa. This relationship suggests that when an outbreak occurs within a smaller time period there is a faster rate of distribution of the disease across space. A good example of this feature may involve what happens when a new serotype is introduced into a new location. This observed dengue epidemiologic pattern is quite unique and has been suggested as a mechanism of dispersal in many countries but the present study provides for the first time evidence for this dengue dispersal pattern and association with transportation hubs in space and time for Trinidad.

During this study DHF cases were observed clustered in space and time (Knox test) and this pattern is similar to that observed in Florida, Puerto Rico [4]. The space-time clustering of the cases suggested the identification of ‘hot spots’ which is similar to that identified by Levine [23] who used this approach to detect crime waves and crime hot spots in the USA. The adoption of this ‘hotspot’ approach has clearly graphically identified areas with the highest dengue activity and once introduced can be an invaluable tool for the analysis of disease dispersal and diffusion from one community to another, for infectious diseases, as well as dengue.

It is also evident that during an epidemic there is a greater probability of infection in areas near and within cities where previous infections or outbreaks had occurred (Table 4), e.g. clusters are frequently observed along the Eastern Main Road (highway running from East to West in the northern part of the island) as shown in Figure 4. This observed pattern explains the emergence of DHF outbreaks in Trinidad which usually occur when there are co-circulating dengue antibodies within a population, generally the leading risk factor for the development of DHF/DSS within clusters in these townships [6]. This circulation pattern explains the increase in DHF cases especially in Trinidad where all 4 dengue serotypes are now endemic [28] and can possibly lead to a massive epidemic in the future with high morbidity and mortality rates.

The present results show that DHF distribution is correlated with transportation hubs as indicated by significant clustering in and around these travel centers. These findings extend the work conducted by Mahabir et al. [5] who identified areas around less voluminous road networks as conducive locations for outbreaks of DHF in Trinidad, especially those associated with or connected to major transportation hubs. Although dengue fever is considered a residential disease its dispersal has been found to be limited to movement of infected people within and outside their home environment [28,39], house to house human movement [12], dispersal of infected *Aedes aegypti* mosquitoes based on the type of housing patterns and type of road network available in the location [5] and the association with travel hubs (present study). Conversely, areas of high traffic volumes were found to limit the movement of mosquitoes [40] and therefore limit the transmission of dengue fever to certain parts of Trinidad [5].

Additionally, there appears to be a distinct association between dengue clustering and types of transportation hubs between epidemic years. During an epidemic year DHF cases are clustered tightly around major city hubs. As the epidemic becomes less intense clusters are found at smaller transportation hubs where smaller cities and towns exist. The reverse effect is seen when transitioning towards an epidemic period. These epidemics occur especially in areas in and around transportation hubs and near cities, which support large commuting and residential populations. These hot spot areas provide suitable breeding habitats and blood meals for Ae. *aegypti* mosquitoes. These results further suggest that the *Ae. aegypti peak* feeding times coincides with high traffic commute times to and from these hubs, that is, during the late afternoon and early morning periods [41]. It is therefore expected that *Ae. aegypti* mosquitoes that feed in these environments will stay in and around these sites since access to blood meals are regularly available for completion of their gonotrophic cycle. Consequently, this results in the rapid growth of mosquito populations along with possible intensification of dengue transmission at these hubs. This may explain the observed dengue transmission patterns at home and at travel hubs and may be responsible for the apparent relationship found between the distance in both space and time between DHF cases: as the number of days decreases between appearance of clusters; the distance between cases increases and vice versa (see Figure 5). This relationship suggests that when an outbreak of disease occurs within a smaller time period than in previous outbreaks, there is a faster rate of geographic dispersal of the disease. In addition, this finding suggests that areas around transportation hubs are suitable environments for the spread of dengue fever. In these environments, people live in close proximity to one another and provide suitable breeding sites for the immature stages and resting sites (indoors and outdoors) for adult *Ae. aegypti* mosquitoes [42]. It is well established that large populations tend to flock around city areas and in close proximity to transportation terminals or routes due to available job opportunities and for quick access to other locations. It is expected that Ae. *aegypti* mosquitoes feeding in these environments will not move very far because of the regular supply of blood meals and artificial breeding sites (8). However, the movement of DF/DHF to smaller towns and cities during an epidemic can lead to outbreaks especially where less emphasis may be placed on vector control thus providing suitable conditions for the establishment or renewal of dengue transmission.

## Conclusions

In conclusion, the results of this study can be used by public health officials to develop maps which can aid in the presentation, visualization and understanding of the geographic distribution of DF/DHF cases, as well as demonstrate trends in the spatial and temporal patterns of reported DF/DHF cases. The clusters observed near and within travel hubs can also be used for developing health education and community awareness programs. It is clear that the current dengue space-time patterns and ‘hotspots’ detection method can be used to help public health officers plan more effective control programs and provide ‘early warning’ alerts with better emergency responses thus allowing better allocation of resources. In 2008–2009 this approach was adopted in Trinidad and significantly reduced the incidence of dengue but was unfortunately discontinued due to logistic and management issues. This component is absolutely vital to program planners since there is already evidence of *Ae. aegypti* resistance to organophosphate and pyrethroid insecticides in the Caribbean [43]. Therefore, in the future, it would be helpful if governments within the Caribbean region could improve their data collection and recording systems for dengue and other diseases to allow time series analyses and thus enable early detection, analysis of epidemiologic patterns, and the management of outbreaks in a more efficient and timely manner.

## Competing interests

All authors declare that they have no competing interests.

## Authors’ contribution

KDS, RSM, JMS, DDC conceptualized the study, conducted field work, conducted data analysis and manuscript preparation, KDC assisted in the data analysis and preparation of manuscript and JBA provided logistic support and assistance for the field study. All authors read and approved the final manuscript.

## Authors’ information

KDS, Ministry of Health, 63 Park Street, Port of Spain, Trinidad, West Indies, RDM and KDC, Department of Geography and Geoinformation Science, George Mason University, Fairfax, Virginia, USA, JMS,JBA and DDC, Department of Life Sciences, The University of the West Indies, St. Augustine, Trinidad and Tobago.
